# Hippocampal and Retrosplenial Goal Distance Coding After Long-term Consolidation of a Real-World Environment

**DOI:** 10.1093/cercor/bhz044

**Published:** 2019-02-20

**Authors:** E Zita Patai, Amir-Homayoun Javadi, Jason D Ozubko, Andrew O’Callaghan, Shuman Ji, Jessica Robin, Cheryl Grady, Gordon Winocur, R Shayna Rosenbaum, Morris Moscovitch, Hugo J Spiers

**Affiliations:** 1 Institute of Behavioural Neuroscience, University College London, London, UK; 2 School of Psychology, University of Kent, Canterbury, UK; 3 Department of Psychology, SUNY Geneseo, Geneseo New York, NY, USA; 4 Institute of Cognitive Neuroscience, University College London, London, UK; 5 Department of Psychology, University of Toronto, Toronto, ON, Canada; 6 Rotman Research Institute, Baycrest Centre, University of Toronto, Toronto, Canada; 7 Department of Psychology, York University, Toronto, Canada; 8 Department of Psychology, Trent University, Peterborough, Canada

**Keywords:** consolidation, hippocampus, long-term memory, navigation, retrosplenial cortex

## Abstract

Recent research indicates the hippocampus may code the distance to the goal during navigation of newly learned environments. It is unclear however, whether this also pertains to highly familiar environments where extensive systems-level consolidation is thought to have transformed mnemonic representations. Here we recorded fMRI while University College London and Imperial College London students navigated virtual simulations of their own familiar campus (>2 years of exposure) and the other campus learned days before scanning. Posterior hippocampal activity tracked the distance to the goal in the newly learned campus, as well as in familiar environments when the future route contained many turns. By contrast retrosplenial cortex only tracked the distance to the goal in the familiar campus. All of these responses were abolished when participants were guided to their goal by external cues. These results open new avenues of research on navigation and consolidation of spatial information and underscore the notion that the hippocampus continues to play a role in navigation when detailed processing of the environment is needed for navigation.

## Introduction

Understanding how the brain consolidates memories is a central question in neuroscience (Mcgaugh 2000). Historically, research has focused on contextual and recognition memory in rodents and primates (Frankland and Bontempi 2005; Eichenbaum et al. 2007; Wang and Morris 2010) and episodic memory in humans (Tulving and Markowitsch 1998; Squire and Wixted 2010; Moscovitch et al. 2016). Despite substantial interest in the neural circuits that support navigation, there has been little systematic investigation directly comparing consolidated spatial memories and representations of environments learned long ago (“familiar” environments) with those learned recently (Rosenbaum et al. 2001; Spiers and Maguire 2007a; Winocur et al. 2010). This dearth of research is particularly surprising considering that current theories disagree about the contribution of the hippocampus to processing spatial representations over time: the standard consolidation theory (SCT) argues that initially the hippocampus is involved in processing the spatial memories, and that over time the representations in neocortical regions are strengthened, reducing the demand on the hippocampus (Squire 1992; Squire and Zola-Morgan 1998). By contrast multiple trace theory (MTT), and its offspring trace transformation theory (TTT), argue that for detailed spatial memories and representations the hippocampus is always involved, or more specifically that an episodic hippocampal trace will exist in addition to the schematized representation in the cortex that can be activated depending on task requirements (Nadel and Moscovitch 1997; Vargha-khadem et al. 2001; Moscovitch et al. 2005; Winocur et al. 2010; Winocur and Moscovitch 2011).

Neuropsychological evidence indicates that complex spatial memories acquired years in the past can become independent of the hippocampus (Teng and Squire 1999; Rosenbaum et al. 2000, 2005; Maguire et al. 2006; Herdman et al. 2015). Such findings are consistent with both SCT and MTT/TTT as long as the memories are schematic, in the sense that they capture information that is sufficient for navigation, such as distances and directions between locations. In several cases, however, hippocampal damage does appear to lead to impaired spatial memories for those detailed aspects of the environment that enable one to re-experience in rich, perceptual details (Rosenbaum et al. 2000; Maguire et al. 2006; Herdman et al. 2015), consistent with MTT/TTT. Insight from functional magnetic resonance imaging (fMRI) research has been highly limited. Only 2 prior fMRI experiments have examined navigation of familiar environments. One study involving London taxi drivers navigating a virtual simulation of London (UK) reported that the hippocampus is engaged at the start of navigating this highly familiar environment (Spiers and Maguire 2006). The other study involved residents of Toronto mentally navigating this city and found no increased activity in the hippocampus (Rosenbaum et al. 2004). Crucially, however, neither study directly compared navigation in familiar with recently learned environments. In a subsequent longitudinal, fMRI study, Hirshhorn, Grady, Rosenbaum, Winocur, and Moscovitch (2012) showed that participants who were newly arrived to a city, initially relied on the hippocampus for navigation but after 6 months could navigate without evidence of hippocampal involvement. Thus, although there is evidence from fMRI that the hippocampus is less implicated in mentally navigating in familiar environments, exactly what the contribution of the hippocampus and other structures is and the nature of their computations is not known. Additionally, as these studies have relied on static, mental navigation, they may not engage hippocampal activity as would active, dynamic navigation in a virtual reality environment. Thus, for now, we cannot rule out whether the differences in hippocampal findings relate to the demands of navigating different cities, with London placing greater demands on mental simulation of future familiar routes than Toronto, or whether the structure of the environment is key to these differences (Spiers and Maguire 2007b).

One question not yet addressed is whether long-term consolidation changes the spatial information processed by brain regions during the navigation of an environment. In recently learned environments, the hippocampus has been shown to encode the distance to the goal (Spiers and Maguire 2007b; Viard et al. 2011; Sherrill et al. 2013; Howard et al. 2014; Chrastil et al. 2015; Balaguer et al. 2016; Spiers et al. 2017) and future goal states (Brown et al. 2016). It is unknown whether this is also the case in highly familiar environments, though studies of static, mental navigation in patients with lesions suggest that their ability to estimate distances and direction to goals is relatively well-preserved, consistent with both SCT and MTT (Teng and Squire 1999; Rosenbaum et al. 2000, 2004; Maguire et al. 2006; Herdman et al. 2015). Current models argue that through systems consolidation, neocortical regions may come to code such information in extensively learned environments (Spiers and Maguire 2007b). Candidate neocortical regions include the retrosplenial cortex (Takahashi et al. 1997; Epstein et al. 2007), parahippocampal cortex (Rosenbaum et al. 2004; Bohbot et al. (2015), for extensive reviews see Spiers and Maguire 2007b; Epstein 2008; Ranganath and Ritchey 2012; Miller et al. 2014), and the anterior cingulate cortex (Teixeira et al. 2006). It is also possible that the involvement of brain regions will vary depending on how complex the future route is and whether individuals plan their route or use certain strategies, with MTT/TTT predicting that the hippocampus, and in particular the posterior hippocampus, would play a more important role if detailed processing is required (Poppenk et al. 2013).

Here we combined fMRI and a virtual simulation of 2 university campuses to examine the brain regions coding the distance to the goal in highly familiar and recently learned environments within the same scan session. Students from 2 London universities (University College London and Imperial College London) navigated each campus, with the one they were not attending made familiar via training material and a walking tour days before the fMRI session. During the scanning session, they engaged in active navigation towards goal locations and subsequently reported on when they planned their routes. We aimed to test whether 1) the hippocampus changes its activity in relation to distances to the goal, 2) this would depend on which environment navigation was taking place in, and 3) how these representations were related to route planning. If schematic representations of distance and direction are used, both MTT/TTT and SCT predict that hippocampal involvement, present in the less familiar environment, will be diminished or absent in the highly familiar environment unless there is a demand on processing a detailed route. Because previous studies comparing navigation in recent and familiar environments did not examine the computations that these representations enabled, it is necessary to determine which of them, and under what conditions, engage the hippocampus. Last, because previous studies relied on static, mental navigation, it is possible that when navigation in a virtual environment is dynamic, it would continue to rely on the hippocampus even in the familiar environment.

## Methods

### Participants

Students from the University College London (UCL) and Imperial College London campuses participated in this experiment. Recruitment involved selecting students who had been studying at either campus for a minimum of 2 years, and had little or no familiarity with the other university campus. This was assessed in a screening interview, in which participants had to label street names and landmarks on a blank map of the campuses. Given the strict rules for recruitment and the large time-commitment, we aimed to collect a sample size similar to our previous study, Howard et al. 2014. We collected 26 datasets, but one participant was excluded due to below chance performance during the fMRI session, resulting in the final sample of 25 subjects (12 UCL and 13 Imperial; mean age: 23 years, range: 20–26; 12 males [5 UCL,7 Imperial], 13 females [7 UCL, 6 Imperial]). Participants were administered 2 questionnaires regarding their navigation abilities and strategies (Santa Barbara Sense of Direction Scale [SBSDS] (Hegarty et al. 2002) and Navigational strategies questionnaire [NSQ], developed in Toronto by J.D.O. and J.R.).

All participants had normal or corrected to normal vision, reported no medical implant containing metal, no history of neurological or psychiatric condition, color blindness, and did not suffer from claustrophobia. All participants gave written consent to participate to the study in accordance with UCL research ethics committee and the Birkbeck-UCL Centre for Neuroimaging (BUCNI) ethics committee. Participants were compensated with a minimum of £70 plus an additional £10 reward for their good performance during the scan.

### Training

**Figure 1:**
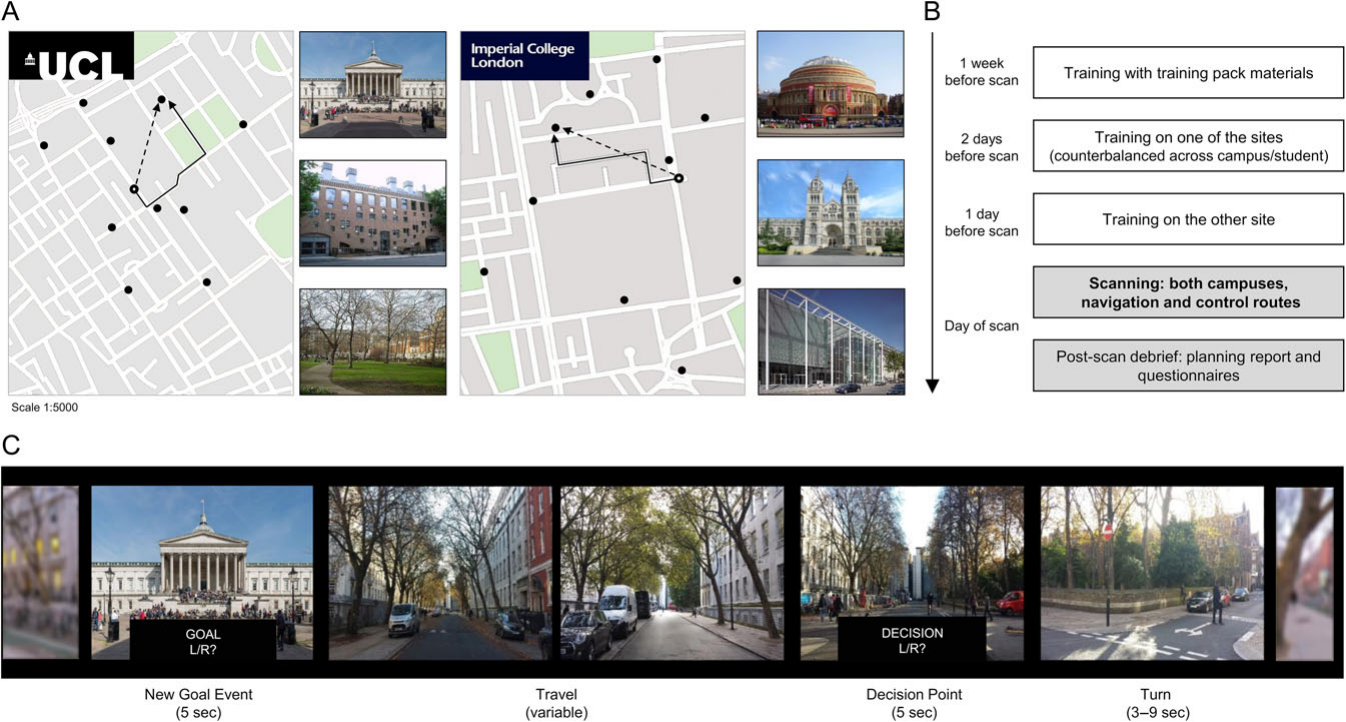
Campus layouts and training protocol. (*A*) Participants navigated around either UCL (left) or Imperial College campus (right), finding their way to various goal locations (examples depicted to the right of the maps—note not all goal locations were necessarily famous). The maps show all potential goal locations marked with black circles. The open circle represents an example participant location, the solid and dashed black lines indicate the optimal path to be taken (Path Distance), and Euclidian distance (“as the crow flies”), respectively, to the current goal location. (*B*) The training protocol consisted of 2 stages. First, a self-guided learning of landmarks and campus layouts with printed training materials. Two days before the fMRI scan participants were taken on an intensive guided tour of one of the campuses, and the next day, they were taken around the other campus. The order was counterbalanced across subjects and could start at either their home campus or the new one. On the day of scanning, participants completed routes in both campuses, in both active navigation and a control condition that involved following directions. During the debrief session, they filled out questionnaires regarding navigation strategies, and performed a behavioral task where they indicated where along the route they engaged in planning while navigating. (*C*) Excerpt from a “navigation” route at UCL. At the start of every route, participants were shown via text on the screen the street they are on, as well as their cardinal facing direction. Next they were given a New Goal Location, and subsequently asked to indicate the general direction of that landmark. They traveled down the road until they reach a junction (the duration and number of images of this was variable depending on the length of the street), at which point they were asked to indicate the correct turn to take towards the goal (Decision Point). Here, the program automatically advanced on a predefined route, which may have been correct, or it may have been an unplanned detour (occurring less frequently). After a variable interval, a new street was entered and the participant was informed again of their location and facing direction. Note the jittered interval after Decision Points is to allow for separating signals relating to Decision Points and Turns (or Detours). Analysis of fMRI data was constrained to the Travel and Decision Point periods. For fMRI analysis, Travel periods were taken as the midpoint between 2 events, and modeled as a punctate event. For simplicity, the above figure only depicts part of a route, and Travel is shown as 2 frames, but could range between 3 and 48 frames (on average: mean 31 ± 18).

The design of the experiment was based on the navigation task reported in Howard et al. (2014). There were, however, 2 campuses in which participants had to navigate: their native familiar campus (“familiar” environment) and the alternative new campus (“recent” environment). All participants needed to learn 10 goal locations, 18 streets, and 8 start points in both environments. Participants were given training materials to practice for a week before the guided tour and the scanning session. Participants were trained on both the recent and familiar campuses in real life with a guided tour by an experimenter, with a strict set of rules, which were as follows: 1) Each road had to be walked past twice, in both directions, and each goal location had to be visited twice. 2) A probe for the name of each goal location was asked once before each visit (experimenter pointed in the direction of the nearest goal location before it became visible). 3) After arriving at each goal location, its name was read to the participant, and the direction of the start location was also given if the goal location was also a starting point. 4) On 5 occasions, participants were asked to point out the directions of 2 goal locations that they had visited twice (all 10 goal locations were reported). 5) The name of each street was asked twice, while the participant was not on it, before and after visiting it. 6) At the end of training, participants were asked about the directions of 10 goal locations and the names of the streets where they were located. The order in which participants were trained on the campuses was counterbalanced across participants and familiarity, and was done to ensure that the familiar campus was also recently visited in its entirety, thereby removing any confounding effects of just the recency of exposure (rather than the age of the memory itself). See Figure 1 for summary.

### Task Design

The task in the scanner was designed to simulate walking through the campuses, by using panorama images from Google Maps Street View (Google 2014), to allow smooth and continuous navigation (developed by J.D.O.). A large part of Imperial and a small part of UCL campuses were not mapped in Google Maps Street view, and these were substituted by panorama images taken by the experimenters (DMD Panorama, Dermandar S.A.L.). The photographs were taken every 6 m. The latitude and longitude coordinates of each panorama image was extracted from Google Earth (Google 2014) for precision. These images were then incorporated in the program filling the gaps left by Google Maps Street View.

Participants performed 16 routes in the scanner: 8 in the familiar and 8 in the recently learned campus. Half of these were “navigation” blocks, that is, participants had to actively navigate. In the other half, the “control” condition, participants were led along the route and only had to make nonlocation based judgements. The order of routes within campuses was counterbalanced, while either navigation and control conditions, or routes between campuses were interleaved across participants. Each route began with a “New Goal Event” to which they were required to navigate. In the navigation condition, participants were asked the general direction of this New Goal (“Left or Right?”) and in the control condition, participants were asked “Can you buy a drink there?”. Each route had multiple New Goal Locations. The routes (trials) lasted around 3 min each, with an average of 3 NGEs per route (range: 1–6). When a participant arrived at a goal, a New Goal Event is presented, but sometimes they were presented with a New Goal Event en-route to a previously indicated one. Following this screen, the program moved to the next panorama image, along the route. These will be referred to as “Travel Period” events, and travel was kept constant at ~25 km/h. When a junction was reached, participants were asked to choose which direction to go (“Decision Point”: “Left,” “Right,” “Straight” in navigation condition and this was given as an instruction with only one option in the control condition), after which the scene would pan into the next street (“Turns”). As the routes were pre-determined, and the response the participant gave had no bearing on the actual trajectory, sometimes the “Turn” events were in fact “Detours,” in which case a nonoptimal path was taken. These events were infrequent, but were included to mimic real-life situations in which travel plans need to be updated. Please see Figure 1, for example, task structure and timings. There was a break between routes, with a new screen indicating the campus as well as whether it was an active navigation or control (“follow”) block. The total number (on average per subject) of each event type was as follows: Travel = 134, New Goal Events (NGE) = 46, Decision Points (DP) = 107, Turns = 76, Detour = 33. Only about ¼ of these values were present per condition. Therefore, we will focus only on Travel periods and Decision Points, as they have a sufficient number of events (>20 per condition). We opted for such a design to increase ecological validity of the task, which necessitates lower trial numbers per condition, however, the 2 main periods of interest were sufficiently powered. We also subdivided the Travel periods via median split into segments with many upcoming turns (≥3) and those with few (<3), in order to investigate the impact of the complexity of the future route (trial numbers: many upcoming turns: 16.6(±1.05), range: 8–26; few upcoming turns: 9.9(±0.4), range: 5–15).

### Postscan Debrief

Immediately after the scan there was a brief interview. All navigation routes that each participant was tested on were replayed in the same order as in the scanner. Participants were instructed to report what they remembered thinking during the navigation, not what they should have done, and to answer questions posed by the experimenter. At the start of each route they were asked “Were you oriented from the beginning?”—this was during the screen shown at the street entry. After that the experimenter pressed the play button. The navigation automatically paused whenever a New Goal Event appeared. Before and after each junction participants were told the responses made in the scanner and the experimenter would ask “Were you planning the route to the goal at this point during the scanning?”. At detours they were asked “Were you replanning at this point?”. They were also asked if they were lost after detours. To this end, we acquired data at the following events (per familiar and recent campus): oriented, lost, and planning (at New Goal Events, Decision Points, and Detours). Participants were also asked to report any salient memory at any point during navigation. All interviews were audio recorded.

### Spatial Parameters

Path Distance (PD), Euclidian Distance (ED), and Egocentric Goal Direction (EGD) were extracted from the data. For example, PD was calculated by summing the length, in meters, of all the component street sections that made up the optimal route. Spatial parameter values were scaled between 0 and 1, where a value of 0 corresponded to being at the goal and a value of 1 to being at the maximum path distance from the goal. Please see Howard et al. (2014) and Javadi et al. (2017) for detailed information on the calculation of spatial parameters. These parameters were then entered into fMRI analyses as regressors at all event types. Please see Table S1 for correlation between spatial parameters at each event type. The aim was to create routes where the spatial parameters were maximally decorrelated. However, due to the nature and layout of the campuses, there were limits on the flexibility of route design. Therefore, we entered path distance independently as a parametric regressor in our analyses, as this was the main focus of our task. However, we also checked our results when including ED along with PD, to establish the robustness of our findings, even with highly correlated regressors.

### fMRI Scanning and Preprocessing

Scanning was conducted at the Birkbeck-UCL Centre for Neuroimaging (BUCNI) using a 1.5 T Siemens Avanto MRI scanner (Siemens Medical System, Erlangen, Germany) with a 32 channel head coil. Each experimental session lasted around 54 minutes and was separated in 3 parts (each of approximately 15–20 min). Approximately 980 functional scans were acquired per session (depending on routes taken), using a gradient-echo incremental EPI sequence (TR = 3400 ms, TE = 50 ms, flip angle = 90°, 40 slices; slice thickness was 2 mm with a gap of 1 mm, slice tilt = 30°. The field of view was 192 mm, and the matrix size was 64 × 64). The scan was a whole brain acquisition, with 40 slices. Note, we used the same sequence as Howard et al (2014) and Javadi et al (2017), and Javadi et al., but included a larger proportion of the parietal area in the coverage, making it impossible to assess the contribution of the entorhinal cortex to the coding of distance, as was reported in Howard et al. A T1-weighted high-resolution structural scan was acquired after the functional scans (TR = 12 ms, TE = 5.6 ms, 1 × 1 × 1 mm^3^ resolution). Ear plugs were used for noise reduction, foam padding was used to secure the head in the scanner and minimize head movements. Stimuli were projected to the back screen, a mirror was attached to the head coil and adjusted for the subjects to see full screen. All fMRI preprocessing and analysis was performed using SPM12 (Statistical Parametric Mapping, Wellcome Trust, London, UK). To achieve T1 equilibrium, the first 6 dummy volumes were discarded. During preprocessing, we used the SPM segment with 6 tissue classes to optimize normalization. Otherwise, we used all default settings, and we performed slice timing correction. No participants had any abrupt motion changes over 4 mm. Scanning was performed in 3 blocks, and as some events occurred rarely, we had to concatenate the fMRI data. We added a session regressor to indicate the change in scanning block.

### fMRI Analysis

For the fMRI analysis, we built multiple models based on a priori predictions from previous work (Howard et al. 2014). Please see Table S3 for a description of the models, events included and regression parameters (if applicable). Note for parametric modulation models, the event of interest was modeled with the corresponding spatial parameter regressors (i.e., PD, ED, and EGD), but also included the other events in order to fully account for activity relating to stimulation. Additionally, we also included a task block regressor, which indicated whether the task was performed in a familiar or recent environment, and navigation or control. Only the implicit baseline (fixation period) of 17 seconds between routes was not included in the model. For example, when modeling Travel Periods, the model would include all Travel Period events in the 4 conditions (familiarity × navigation) + parametric modulators (pmods), in addition to the other events: DP, NGE, Detours, Turns, and Session, for each of the 4 conditions. Note that Travel Periods were defined as a single point in time while traveling down a route, and so was modeled as a punctate event using a stick function. Small-volume correction was done with defined anatomical masks. The hippocampus mask was a combination of right mid and posterior sections (a priori based on the results of Howard et al. 2014). The retrosplenial mask was based on the anatomical restrosplenial cortex specifically (BA 29/30). We also built global mask involving the sum of a priori regions for remote memory based on the review by Spiers and Maguire (2007b): anterior cingulate cortex, bilateral caudate, bilateral parahippocampal cortex, retrosplenial cortex and the right hippocampus (see Fig. S7 for details).

Note, our parametric analyses of distance were always focused on a-priori regions of interest, given our main question was how coding of distance in the hippocampus may change with memory consolidation. For completeness, we report uncorrected (*P* < 0.001, min. 5 contiguous voxels) whole-brain results for the categorical and parametric effects in the Supplementary Material (Figures S1 and S2, Table S4), to help future studies to reference the full dataset.

## Results

Our experimental fMRI task was adapted from Howard et al. (2014), in which participants (*n* = 25) were presented with a goal location in a virtual simulation of the environment and required to travel along the streets (Travel Periods) and make path choices prior to street junctions (Decision Points), see Supplementary Material and Figure 1. In matched control routes participants were instructed which street to select during navigation to goal locations. Our task differed to Howard et al. (2014) in that there were 2 environments to navigate: a familiar campus (“familiar”) and a new campus (“recent”).

Participants were exposed via an intensive in situ training tour of both campuses in the immediate preceding days before scanning, ensuring that the crucial difference between the 2 environments was the long-term (>2 year) prior knowledge of one of them. In the pre-training assessment, we wanted to confirm that participants knew their own campus well, and were unfamiliar with the other campus. In the familiar environment participants were on average 48% and 86% accurate on street names and landmarks, respectively. Conversely, for the recent campus, they were only 10% accurate on the street names, and 22% for landmarks. Thus, there was a significant effect of environment (*F*[1,24] = 140.8,*P* < 0.001), type of information probed (*F*[1,24] = 48.9,*P* < 0.001), and interaction (*F*[1,24] = 14.8, *P* = 0.001). Post hoc paired *t*-test were also all significant (all *t* > 3, *P* < 0.004), underscoring the notion that participants did indeed have better knowledge about their own campus, and mainly its landmarks.

Behavioral results revealed that participants were able to orient themselves and make correct decisions at Decision Points in both environments, albeit better so in their familiar campus (Familiar: *M* = 89.9%, SD = 11%; Recent: *M* = 80.4%, SD = 13%, paired sample *t*-test: *t*[24] = 3.6, *P* = 0.002). Participants responded more quickly in their familiar environment (Familiar = 1.19 ± 0.4 s, Recent = 1.39 ± 0.5 s, *t*[24] = −4.7, *P* < 0.001), and reaction times scaled with distance to the goal along the future path (across subjects and junctions, both environments *r*[124]>0.24, *P* < 0.01, Table S2). To control for potential differences in brain activation due to reaction time differences in the familiarity condition, we included a trial-by-trial inverse efficiency score (RT/mean accuracy) as a regressor when modeling the Decision Points.

After scanning participants completed a debriefing session, which consisted of filling out a questionnaire regarding their navigational strategies, as well as a behavioral version of the task, in which they indicated whether they had engaged in planning during Decision Points. This was only done for the active navigation routes. We compared responses to familiar and recent environments and found that participants did report more planning at Decisions Points in the recent environment (familiar: 8%, recent: 22%, *t*<−2.9, *P* < 0.006). We also found that participants who planned more in familiar environments also planned more in recent ones (*r* = 0.6, *P* = 0.001). Relating performance in the scanner to debriefing responses we found that RTs at Decision Points in recent environments correlated with amount of planning at Decision Points (both familiar and recent; *r* = 0.42, *P* < 0.033), such that less planning at DPs meant quicker responses. However, there was no correlation between planning and accuracy (all *r*>−0.16, *P* > 0.1). Finally, we investigated the strategies questionnaires. Participants scored an average of 4.2 (range: 2.7–5.7) on the SBSDS. On the NSQ, they scored an average of 7.2 points (out of 14, where the maximum indicates only map-based navigation strategies). There was no correlation between the scores on these 2 questionnaires, between these scores and performance in the scanner, or to the amount of planning reported. We report exploratory analyses relating brain activity to the planning and navigational strategies in the Supplementary Material.

### fMRI Results

A categorical analysis of different event types and task blocks revealed more activity in lateral and medial parietal areas in familiar environments when actively navigating (“navigation” condition) compared with just following along a route (“control” condition), whereas recent environments did not show such a distinction (Fig. S1). There were no clear differences when comparing familiar to recent navigation (Fig. S2 and Supplementary Results). In order to examine how the neural responses relate to metrics in computational models we interrogated our fMRI data with parameters related to the distance and direction to the goal. Specifically, we explored how path distance to the goal (Fig. 1*A*) was correlated with brain activity during events sampled during Travel Periods and at Decision Points. We focused on these events because we were able to sample >20 events per condition (familiar/recent, navigation/control). Small-volume correction (*P* < 0.05) was performed using anatomical masks (as described in detail in the Methods section). All results reported are displayed at *P* = 0.005 uncorrected (for completeness, a full list of significant voxels reported at *P* < 0.001, min. 5 contiguous voxels in Supplementary Table S4). A global mask involving the sum of a priori regions for remote memory did not reveal any significant clusters, but targeted ROIs for hippocampus and retrosplenial cortex did reveal significant effects and are reported below. From here onwards, the term “distance” refers specifically to “path distance” (see Methods for discussion on ED).

### Right Hippocampal Activity is Correlated With Distance to the Goal During Travel Periods of Navigation in Recently Learned Environments and When Many Turns Lie Ahead in Familiar Environments

**Figure 2.**
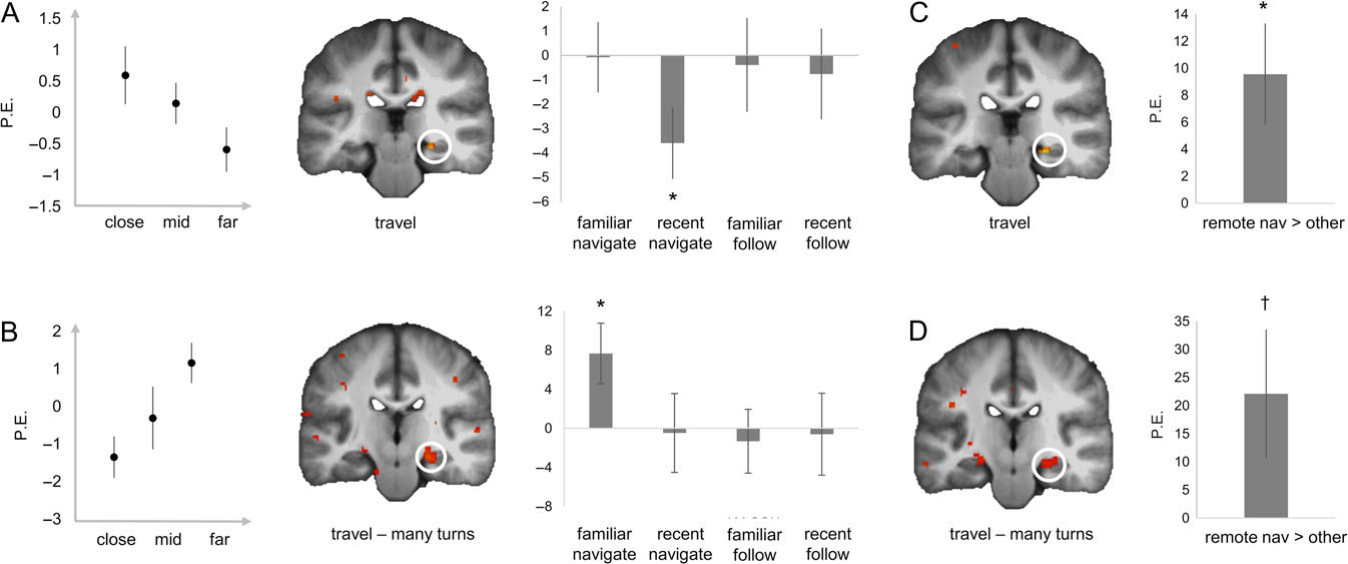
The hippocampus is involved in processing path distance in novel environments as well as familiar environments when the route is complex. (*A*) During Travel in recent environments, there was a significant negative correlation with path distance, such that there was higher BOLD activity in the hippocampus when participants were closer to the goal location. (*B*) During Travel in familiar environments, there was a significant positive correlation with path distance, such that there was higher BOLD activity in the hippocampus when participants were further away from the goal location, but only when the route encompassed many turns (>=3). In each plot, from left to right: parameter estimates (PE) extracted from a categorical model (binned by distance), the BOLD activity for the relevant condition (displayed at *P* < 0.005, min. 5 contiguous voxels), and the PE from the peak voxel in the ROI for each condition, for illustrative purposes only (note the “*” refers to SVC of the voxels in the SPM; error bars represent s.e.m.). All effects survive small-volume correction, including when ED (Euclidian distance) is added to the models. (*C*, *D*) Show the data when the GLM included weighted regressors for the effects seen in *A*/*B*, respectively. For example, in *C*, the contrast was 1 −3 1 1, testing for an overall effect of correlation with path distance during Travel, for the recent navigation condition compared with all other conditions. *P < 0.05 SVC, ^†^*P* < 0.1 small-volume correction (SVC).

During Travel Periods when navigating the recently learned environment, we found a significant negative correlation with the distance to the goal in the right midposterior hippocampus (Fig. 2*A* and Table S4), indicating that hippocampal activity increased with proximity to the goal. This effect was absent in the familiar environment and the control routes (Fig. 2*A*, right panel). Moreover, right posterior hippocampal activity was significantly more correlated with proximity to the goal during navigation in the recently learned environment than in the other conditions combined (Fig. 2*C* and Table S4).

Because MTT/TTT argues that the posterior hippocampus is involved in processing detailed information even after extensive consolidation (Moscovitch et al. 2005) we examined whether the hippocampal activity might code the distance to the goal when the route ahead required the consideration of many possible turns (using a median spilt). For Travel periods with many upcoming turns (≥3) in the familiar environments there was a significant hippocampal cluster, positively correlated with path distance in the Travel events with many turns (Fig. 2*B*). There was no significant correlation between hippocampal activity and path distance when there were few turns (<3) in familiar environments or either type of Travel event in the recent environments or during the control routes. Thus, when navigating highly familiar environments and the route ahead will require multiple decisions the hippocampus appears to be engaged in coding the distance to the goal.

### Retrosplenial Cortex Codes Distance to the Goal During Travel in Familiar Environments

**Figure 3.**
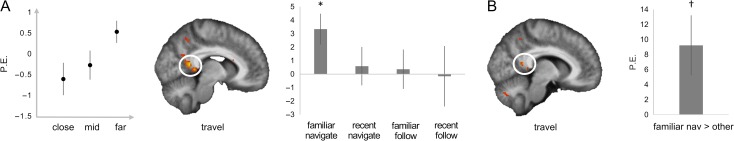
The retrosplential cortex codes path distance after consolidation. (*A*) During Travel in familiar environments, there was a significant positive correlation with path distance, such that there was higher BOLD activity in the retrosplenial cortex when participants were further away from the goal location. In each plot, from left to right: parameter estimates (PE) extracted from a categorical model (binned by distance), the BOLD activity for the relevant condition (displayed at *P* < 0.005, min. 5 contiguous voxels), and the PE from the peak voxel in the ROI for each condition, for illustrative purposes only (note the “*” refers to SVC of the voxels in the SPM; error bars represent s.e.m.). All effects survive small-volume correction, including when ED (Euclidian distance) is added to the models. (*B*) Shows the data when the GLM included weighted regressors for the effects seen in A, such that the contrast was −3 1 1 1, testing for an overall effect of correlation with path distance during Travel, for the familiar navigation condition compared with all other conditions. ^†^*P* < 0.1 SVC.

During Travel Periods of navigation routes in the familiar environment we observed a significant positive correlation with distance in the retrosplenial cortex (Fig. 3 and Table S4), indicating the activity was greatest when participants were farthest from their goal. This response was absent in the recent environment and during the control conditions (Fig. 3*A*, right panel). Retrosplenial activity was significantly more correlated with the distance to the goal in familiar navigation routes than in the other conditions combined (Fig. 3*B* and Table S4). Even at liberal thresholds, activity in our other predicted ROIs (anterior cingulate cortex, caudate and parahippocampal cortices) was not significantly correlated with distance to the goal during Travel Periods in either recent or familiar environments (Fig. S3).

### Control Analyses for Effects of Path Distance During Travel Periods

To further scrutinize the data, we conducted several control analyses. We included ED in the models investigating parametric effects of PD. We replicated the PD results, emphasizing the robustness of these effects (see Table S4), despite highly correlated parametric regressors (see Table S1). As our original Travel analysis included both travel midpoints as well as New Street Entry events, we also examined the data with 2 additional models, which separated these events. This was to remove any concern that the results found during Travel events were not contaminated by entry into a new street, as these may be considered as “sub-goal” points that are informative as to the position/segment along the route. When examining Travel midpoints only, we replicated the right hippocampal effect in recent navigation, though this only survived a mid-hippocampal SVC (Table S4), however we did not replicate the retrosplenial cortex in familiar navigation routes, but there was a positive correlation of PD in the precuneus and parietal–occipital sulcus (Table S4). Both the hippocampal and retrosplenial effects were absent from the New Street Entry only models, emphasizing that the correlation with PD was specific to the Travel periods.

We also examined whether there was any evidence that the negative correlation with PD coding in the hippocampus was related to approaching the goal when it was straight ahead. We modeled Travel periods when this was the case, point-by-point, for segments that were at least 7 s long (minimum 2 TRs, which was 3.4 s). Two subjects did not have segments long enough (this depended on the routes they were given and some routes had shorter “goal approach” segments) and were not included in the analysis. We did not find any evidence for coding in the hippocampus for recent navigation, which may be due to the reduced power (overall fewer samples), or perhaps that given the visualization of the task, the goal was not actually visible on each panorama image and as such it wasn’t as clear as it would have been if it were a continuous movie stream. In addition, we also ran a model in which the number of upcoming turns was included as a parametric regressor for Travel (instead of PD), and found no effects in either recent or familiar environments.

### Spatial Coding at Decision Points

Because of recent evidence of goal-related egocentric and proximity combined modulation of activity in the hippocampus at Decision Points (Howard et al. 2014) and during flight by bats (Sarel et al. 2017), we also explored whether there was any such modulation at Decision Points. During the navigation task in recent environments we found the mid hippocampus was more active the further away, and less directly ahead the goal was (see Table S4 for details of activation). We also found more generally that the hippocampus was more active when the goal was far away at Decision Points during navigation of recent environments (see Supplementary Results, Fig. S6). Note egocentric goal direction was also highly correlated with path distance (Table S1).

## Discussion

Using fMRI combined with a virtual simulation of 2 London university campuses, we examined how, during navigation, the distance to the goal is represented by brain regions when the environment is highly familiar or recently learned. We found that right posterior hippocampal activity was correlated with the distance to the goal in recent environments and in also in familiar environments when the route ahead required many turns. By contrast we found the retrosplenial cortex only tracked the distance to the goal in familiar environments. These results help inform debates about memory consolidation, navigation systems of the brain, and the functional differentiation of the long-axis of the hippocampus.

### Representation of the Distance to the Goal: Hippocampus

The correlation of hippocampal activity to distance to goal during travel periods in navigation routes in the recently learned environment agrees with prior fMRI reports of similar coding (Viard et al. 2011; Sherrill et al. 2013; Howard et al. 2014; Balaguer et al. 2016) and supports models which argue the hippocampus computes information about the future path to goal for navigational guidance (Erdem and Hasselmo 2012; Penny et al. 2013; Bush et al. 2015). The observation that this was specific to navigation routes and not present in control routes is consistent with prior evidence (Howard et al. 2014) that such distance tracking is not automatic and requires goal-directed navigation. It is possible that the hippocampal activity correlated with distance to the goal relates to the preactivation of place cells along a route to the goal, or “forward-sweeps” of place cell activity towards the goal (Spiers and Barry 2015). However, such responses may also relate to the recently discovered path distance-coding neurons in the CA1 hippocampal region of flying bats (Sarel et al. 2017), where each distance-coding cell expresses activity at a certain distance from the goal and more cells are active near the goal, which would give rise to an fMRI signal that changes with goal-distance.

Consistent with our previous study exploring distance coding in a recently learned real-world environment (Howard et al. 2014), we found the posterior right hippocampus coded distance to the goal during travel periods in recently learned environments. However, while Howard et al. (2014) found a positive correlation between posterior right hippocampal activity and distance, we observed a negative correlation in the recently learned environments. Such a discrepancy in the direction of the correlation has also be observed across several other fMRI studies (see Spiers and Barry 2015). A probable determinant of the relationship between hippocampal activity and goal distance is variation in the structure of the environment. For example, an environment with many intersections would likely require more planning and retrieval of the space, placing greater demands on the neural regions responsible for coding the path to the goal when far from the goal. Consistent with this, we found that when the future route required many turns and was in a familiar environment, a positive correlation with hippocampal activity and the distance was observed. Notably, the environment used by Howard et al. (2014) (Soho, London, UK) contained more junctions to navigate across compared with the environments we used, thus most routes examined would likely have required many turns. Another difference between the environments that may have been important was the amount of visual detail in the environments. Soho contains many more distinct landmarks than the campuses we used. If the positive correlation with distance is linked to retrieval of details on the route ahead it may be that it took longer for participants to acquire a similar level of detail in the university campuses than it would for Soho, resulting in a similar pattern of goal coding across environments only once sufficient knowledge has been acquired. Consistent with this argument, prior fMRI studies using recently encountered environments containing less perceptual detail found that the hippocampal activity increased with proximity to the goal (Viard et al. 2011; Sherrill et al. 2013). It is also important to consider that the overall size of the environments differed between our study (up to 1 km) and Howard et al. 2014 (400 m), which may also play a role in driving different representations of goal-distance. This may also underlie why we observed increased right posterior hippocampal activity at decision points when the goal was far away in the recent environments. Recent evidence indicates that turns in a path and the familiarity with an environment can also impact on representations of distance (Bonasia et al. 2016; Jafarpour and Spiers 2016; Brunec et al. 2017). Thus, it will be important in future work to specifically manipulate these different variables and map distortions in spatial memories when examining goal-distance coding.

### Representation of the Distance to the Goal: Retrosplenial Cortex

It has remained unclear which brain regions are responsible for spatial processing in highly familiar environments (Spiers and Maguire 2007b). Several amnesic case studies have displayed impressive retention of spatial memories for environments learned before the onset of extensive hippocampal damage (Teng and Squire 1999; Rosenbaum et al. 2000; Maguire et al. 2006; Herdman et al. 2015). One brain region posited as supporting this long-term knowledge has been the retrosplenial cortex (Spiers and Maguire 2007b). Here we find evidence to support this proposal, revealing that the retrosplenial cortex specifically tracks the distance to the goal when the environment is familiar, but not when it has only recently been learned in the preceding days. Like the hippocampus, we found retrosplenial cortex only tracked the distance to the goal when participants were required to use their memory to navigate, not when they were guided with which choices to make at each junction. Our findings are broadly consistent with fMRI evidence that retrosplenial cortex activity is positively correlated with the distance back to the start of a simple circular journey (Chrastil et al. 2015). It is also consistent with electrophysiological evidence in rodent retrosplenial cortex of travel distance coding during a route traversal task (Alexander and Nitz 2017), sequence read-out of “place cell” like responses from retrosplenial cells (Mao et al. 2017) and the development of goal-location coding (Miller et al. 2018).

### Functional Differentiation Within the Hippocampus

Similar to our previous study (Howard et al. 2014), we found a right posterior hippocampal focus for activity correlated with the distance to the goal. Prior neuropsychological research has consistently indicated the dominance of the right hemisphere in processing spatial information (Spiers et al. 2001; Burgess et al. 2001, 2002) and the posterior locus agrees with the view that posterior hippocampus processes fine detail, such as spatial metric information (Poppenk et al. 2013). An important question remains regarding the change in navigational strategies across environments over the course of increased exposure, and how this may alter the neural representation: would more familiarity cause navigation to activate a more coarse anterior–hippocampal representation (Poppenk et al. 2013), or do map-based navigators always activate detailed representations in the posterior hippocampus irrespective of the environment?

### Systems Consolidation of Spatial Representations

Dominant accounts of memory consolidation, SCT, and MTT/TTT, both claim that with the passage of time and experience, performance on some tasks becomes independent of the hippocampus and comes to rely on extra-hippocampal structures such as the retrosplenial cortex (Marr 1971; Alvarez and Squire 1994; Frankland and Bontempi 2005; Winocur and Moscovitch 2011; Sekeres et al. 2018). What distinguishes them is whether the information represented in hippocampal and extra-hippocampal structures, and the computations performed by them are the same, as SCT contends, or are different, as MTT/TTT argues. Our findings favor MTT/TTT. Firstly, we observed evidence for continued hippocampal engagement in an environment learned 2 years prior to testing, which is at odds with SCT. Secondly, we found a transformation in the direction of the correlation between the hippocampus and the distance to the goal during travel periods when familiar environments were compared with recently learned environment. This reversal suggests that the computations and/or the type of information that is represented in the hippocampus changes with consolidation. Finally, in agreement with the proposal of MTT/TTT that the hippocampus would play a greater role in the processing of detailed memories after extensive consolidation, we found that hippocampal activity tracked the distance to the goal in the familiar environments when detailed information about the future route was required.

Our observation that retrosplenial cortex only comes to code the distance to the goal after extended experience with the environment is consistent with several past studies which have observed increased activity in the retrosplenial cortex with the acquisition of spatial knowledge about an environment (Wolbers and Büchel 2005; Auger et al. 2012, 2015). It is also consistent with reports of disorientation in highly familiar environments after retrosplenial lesions (Aguirre and D’Esposito 1999; Maguire et al. 2001; Spiers and Maguire 2007b). It remains to be seen whether this shift is accompanied by a transformation from detailed representations to a more gist-like schema, as has been proposed for the involvement of the retrosplenial cortex in models of contextual memory (Ranganath and Ritchey 2012) and by TTT (Winocur et al. 2010; Winocur and Moscovitch 2011; Sekeres et al. 2018), or alternatively if increased retrosplenial activity in familiar environments is a consequence of shifting between egocentric and allocentric representations during navigation (Marchette et al. 2014) (the latter of which would be potentially less available in recent environments).

## Conclusion

In summary, our fMRI study provides the first comparison of active navigation of both a recently learned and a highly familiar environment. Our data supports models in which there is change in demand on brain regions with extended consolidation of the memories of an environment, with the retrosplenial cortex specifically tracking the distance to the goal in familiar environments and the hippocampus tracking goal distance in recent environments and also familiar environments when the future route contains many turns. Future research will be useful to determine how neuronal-level activity in the hippocampus and retrosplenial cortex may give rise to the fMRI signal dynamics reported here. Additionally, studies designed to examine different types of navigational strategies, including planning across different environments would be useful to help investigate whether hippocampal activity is present when people engage in complex decision-making even in very familiar environments, which might help reconcile the predictions of dominant theories of memory transformation and consolidation.

## Supplementary Material

Supplementary Data
