# Supplementary material for: Hippocampal and Retrosplenial Goal Distance Coding After Long-term Consolidation of a Real-World Environment
Source: Cereb Cortex. 2019 Feb 20;29(6):2748–58. doi: 10.1093/cercor/bhz044 (PMC6519689; doi:10.1093/cercor/bhz044)

Table S1: Correlation between Event Types and raw Spatial Parameters.
Path Distance (PD), Euclidian Distance (ED) and Egocentric Goal Direction (EGD)

EVENT	**PD-ED**	**PD-EGD**	**ED-EGD**
**Decision Point**	0.78**	0.38**	0.19*
**Travel**	0.67**	0.62**	0.26**

**p<0.001;*p<0.05, Bonferroni corrected;

Table S2: Correlation between reaction times at Decision Points and spatial parameters across all subjects (r-values shown)

	**PD**	**ED**	**EGD**	**PDxEGD†**
**OVERALL**	0.31**	0.23*	0.05	0.27**
**FAMILIAR**	0.28**	0.17~	0.02	0.26**
**RECENT**	0.24**	0.21*	0.07	0.22*

**sig. p<0.01,*sig p<0.05,~trend p<0.08,†to compare to Howard et al, 2014

Table S3: Details of GLM parameters for the fMRI models and durations (below)

MODEL	CONDITIONS	ParametricModulators
**CATEGORICAL- all events**	**Environment (2) x Nav (2)**	**IES***
**Travel**	**Environment (2) x Nav (2)**	**PD**
**Decision Point**	**Environment (2) x Nav (2)**	**PD, IES***
CONTROL MODELS		
Travel	Environment (2) x Nav (2)	PD, ED
DP	Environment (2) x Nav (2)	PD, ED, IES*
Travel – distances binned (illustration only)	Environment (2) x Nav (2)	PD
DP – distances binned (illustration only)	Environment (2) x Nav (2)	PD, IES*
Travel – midpoints between events only	Environment (2) x Nav (2)	PD
Travel – New Street entry only	Environment (2) x Nav (2)	PD
Travel – Dead Ahead Goals only (each step modelled)	Environment (2) x Nav (2)	PD
Travel – few turns in segment (<3)	Environment (2) x Nav (2)	PD
Travel – many turns in segment (>=3)	Environment (2) x Nav (2)	PD
Travel	Environment (2) x Nav (2)	Turns (# upcoming)
ACROSS SUBJECTS MODELS		Second-level
Travel	Environment (2) x Nav only	DP planning scores
DP	Environment (2) x Nav only	DP planning scores
Travel PD	Recent Nav**	NSQ mapping scores

*Inverse efficiency: trial-by-trial RT/mean accuracy to control for differences in RT between familiar and recent condition during navigation (see behavioural results below)

**Note that the GLM was run post-hoc after finding a significant correlation using extracted parameter estimates per person (in the peak hippocampal voxel for the Travel PD effect (recent navigate)) and mapping scores.

EVENTS	DURATIONS
Decision Point	5s
New Goal Event	9s
Detour	6s
Turns	6s
Travel	0s
Session	variable

Supplemental Results

Hippocampal activity is positively correlated with the distance to the goal at decision points

At Decision Points during the navigation of the recently learned environment right posterior hippocampal activity was positively correlated with the path distance to the goal (Figure S5, Table S4). This response was observed when including inverse efficiency scores (IES) in the model (see Supplemental Methods), to account for differences in reaction times and accuracy across events. This response was absent in the familiar environment and control routes, and there was also no parametric hippocampal response to IES on its own, underscoring the fact that it was not behavioural differences between environments that was driving the hippocampal response. Right posterior hippocampal activity was more correlated with the distance to the goal in recent navigation routes than in the other conditions combined, at a threshold of $p < 0.005$ uncorrected (Figure S5B). While responses to distance during Travel were relatively linear for both hippocampus and retrosplenial cortex activity (Figure S4A), the hippocampal response during decision-making appears better characterised as categorical engagement at very large distances (Figure S4B).

Given our prior targeted hypotheses, and because we do not aim to compare Decision Points to Travel periods, we did not conduct a full-factorial analysis, which would be 2 x 2 x 2 x 2 (task x environment x period x ROI). When comparing recent and familiar navigation directly, we found a trend for the hippocampal effect at Decision Points after SVC ($p=0.062$), but the hippocampal and retrosplenial effects during Travel for the same comparisons did not survive SVC. This failure to reach standard statistical significance in these contrasts may relate to the reduced power in these specific contrasts compared to Howard et al., 2014.

Individuals who report more route planning in familiar environments have greater activity in their posterior hippocampus during the navigation of familiar environments

According to MTT/TTT and scene construction theory of hippocampal function (Hassabis & Maguire, 2009; Moscovitch et al., 2005), conscious mental simulation of future routes should engage the hippocampus no matter how familiar the environment may be. To test this prediction, we conducted a post-scan debriefing with a video replay of the navigation routes. At each Decision Point and New Goal Event the replay was paused and participants were asked whether they could recall planning their route to the goal. We found participants reported more route planning at events in recently learned environments than familiar environments (New Goal Events, Familiar: M=57%, SD=39; Recent: M=69%, SD=30; Decision Points, Familiar: M=8%, SD=10; Recent: M=22%, SD=20; paired t-test, both $t<-3$, $p<0.007$). Interestingly, in navigation routes in familiar environments, across participants, right posterior hippocampal activity was significantly correlated with the amount of reported planning at Decision Points (Figure S6A Table S4). This same measure of planning was also correlated with hippocampal activity during Travel Periods in familiar environments (Figure S6B, Table S4). We found performance accuracy at Decision Points did not correlate with hippocampal activity during navigation routes, either in the recently learned environment or the familiar environment (familiar and recent environments both: $r<0.3$, $p>0.1$), and that the amount of planning reported was not correlated with performance accuracy (familiar and recent environments both: $r>-.06$, $p>0.1$).

Self-reported ‘map-based’ navigators have stronger correlations between hippocampal activity and the distance to the goal than ‘route-based’ navigators

Prior evidence indicates that strategy use for navigation impacts on the engagement of different brain regions for navigation of simulated environments (Iaria, Petrides, Dagher, Pike, & Bohbot, 2003; Iglói, Doeller, Berthoz, Rondi-Reig, & Burgess, 2010). To test whether this is true for navigation of real-world environments, participants completed a questionnaire probing navigational strategy use (see Supplemental Methods). The questionnaire determines the extent to which a person uses a map-based approach for navigation or a sequential landmark-based approach for navigation. For example, questions may refer to how an individual imagines a route they will take to get to their goal: using a bird’s-eye view constitutes map-based navigation, whereas visualizing buildings and things seen on the way would indicate landmark-based navigation. We were interested if the type of navigational strategy is indicative of how space is represented during active navigation. Participants with higher map-based navigation scores had significantly stronger negative correlations between right posterior hippocampal activity and the distance to the goal during Travel Period in the recently learned environments (Figure S6C). We found no correlation between this self-reported strategy use and the amount of route planning ($r>-0.18$, $p>0.1$).

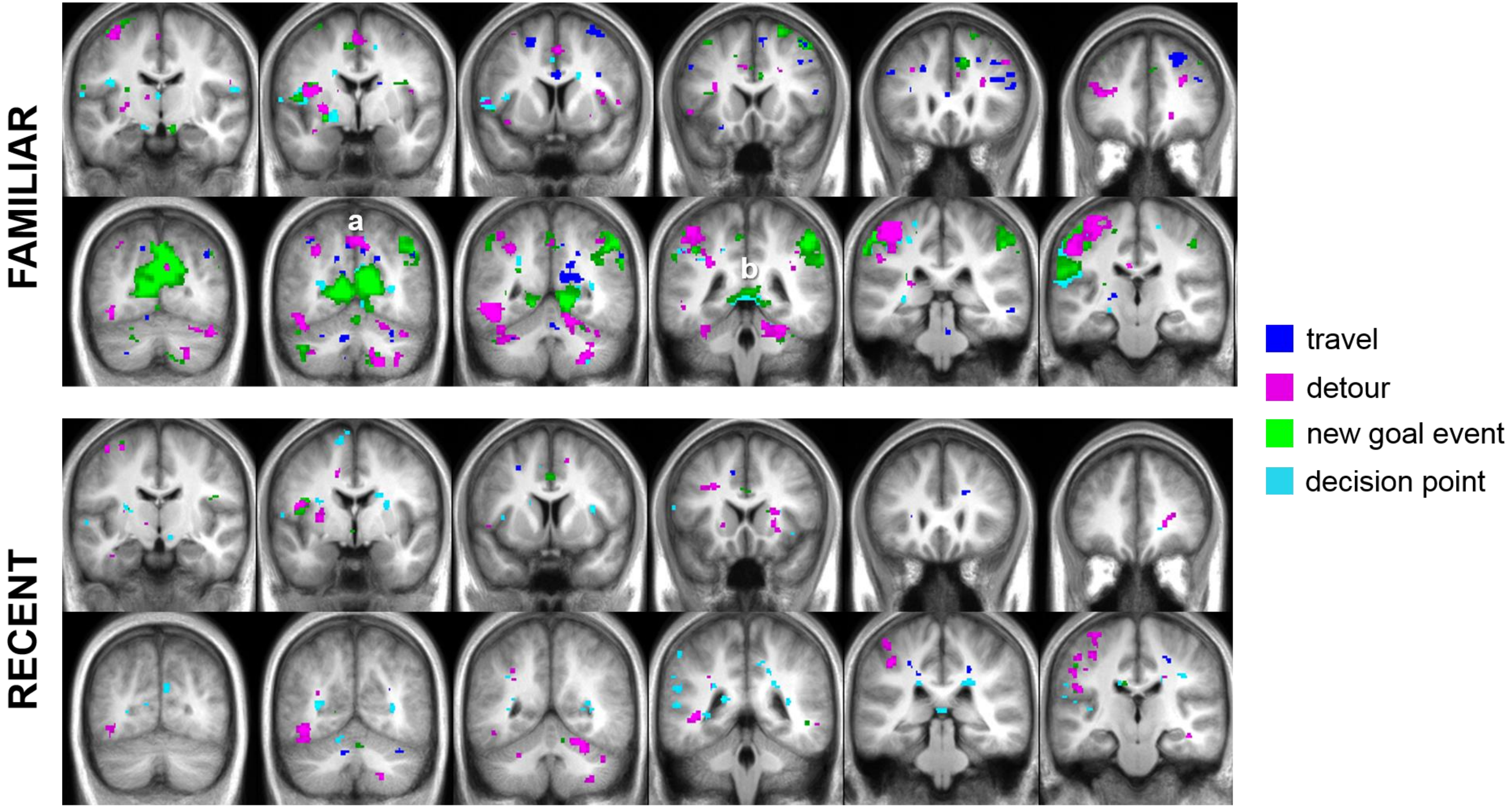

Figure S1: **Categorical effects of all events for both familiar and recent environments (navigate > follow).** a) Precuneus: Travel*, Detour*, and NGE*. b) Retrosplenial: Travel, DP* and NGE*. *p<0.05 small volume correction (SVC). Slices shown are y=-73 to +37, every 10 slices (MNI).

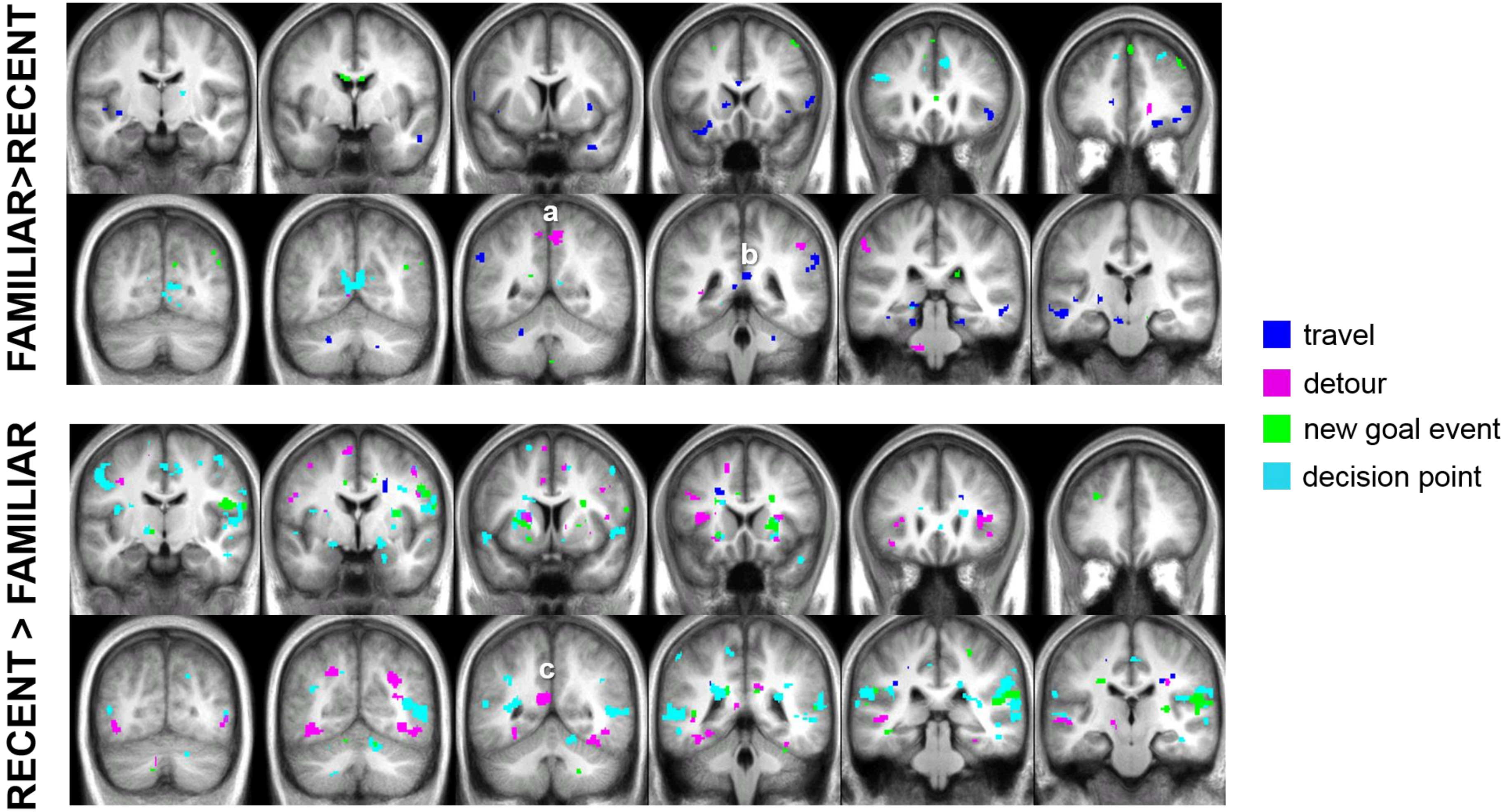

Figure S2: **Categorical effects of all events for familiar > recent (navigation only)** a) Precuneus: Detour* b) Retrosplenial: Travel* c) Detour*; *p<0.05 small volume correction (SVC). Slices shown are y=-73 to +37, every 10 slices (MNI).

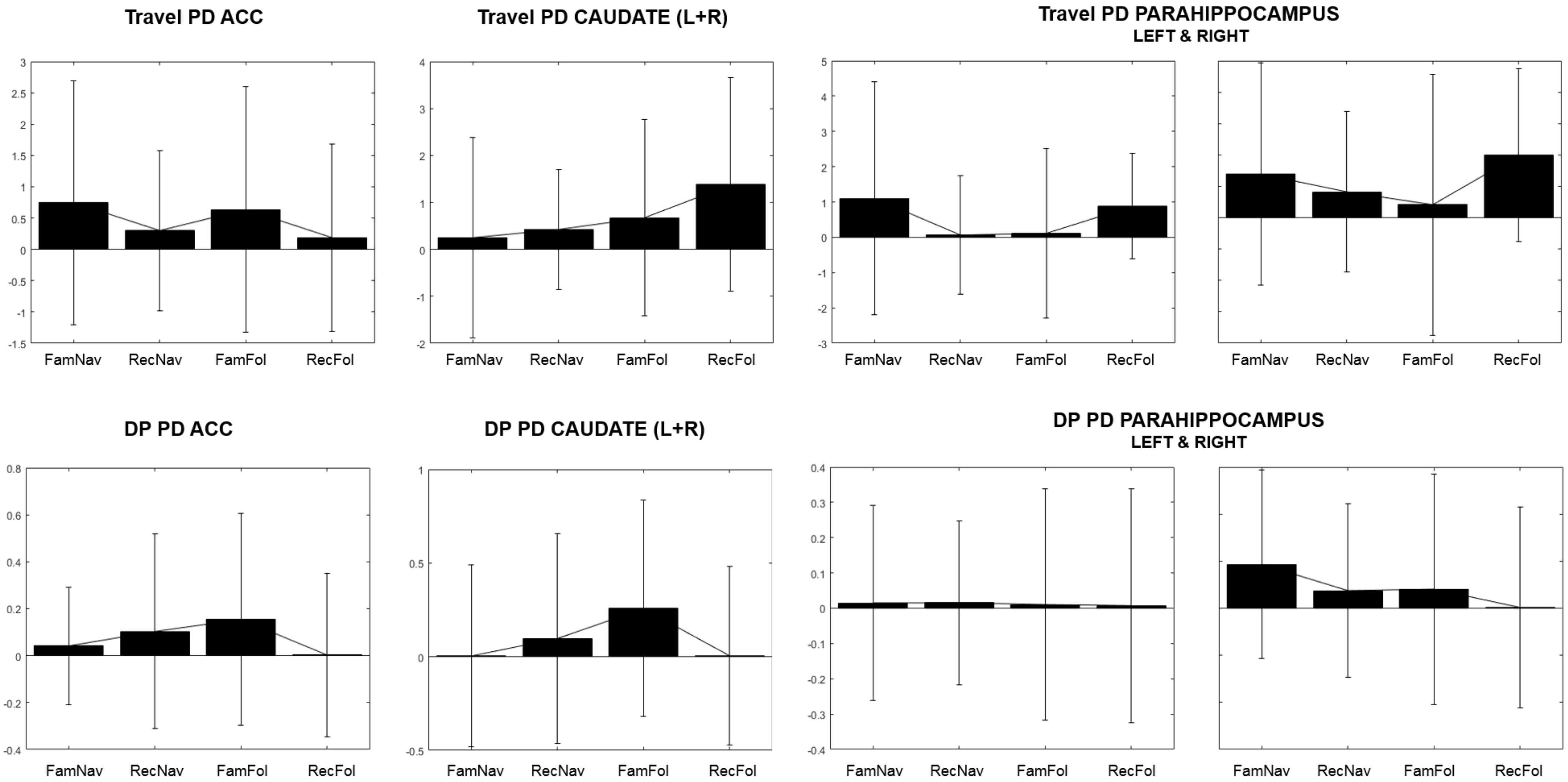

Figure S3: Parameter estimates from a-priori ROIs involved in navigation and memory. None of these areas seen at p=0.005 for any of the contrasts in Fig3/4, and no areas or conditions survive small volume correction.

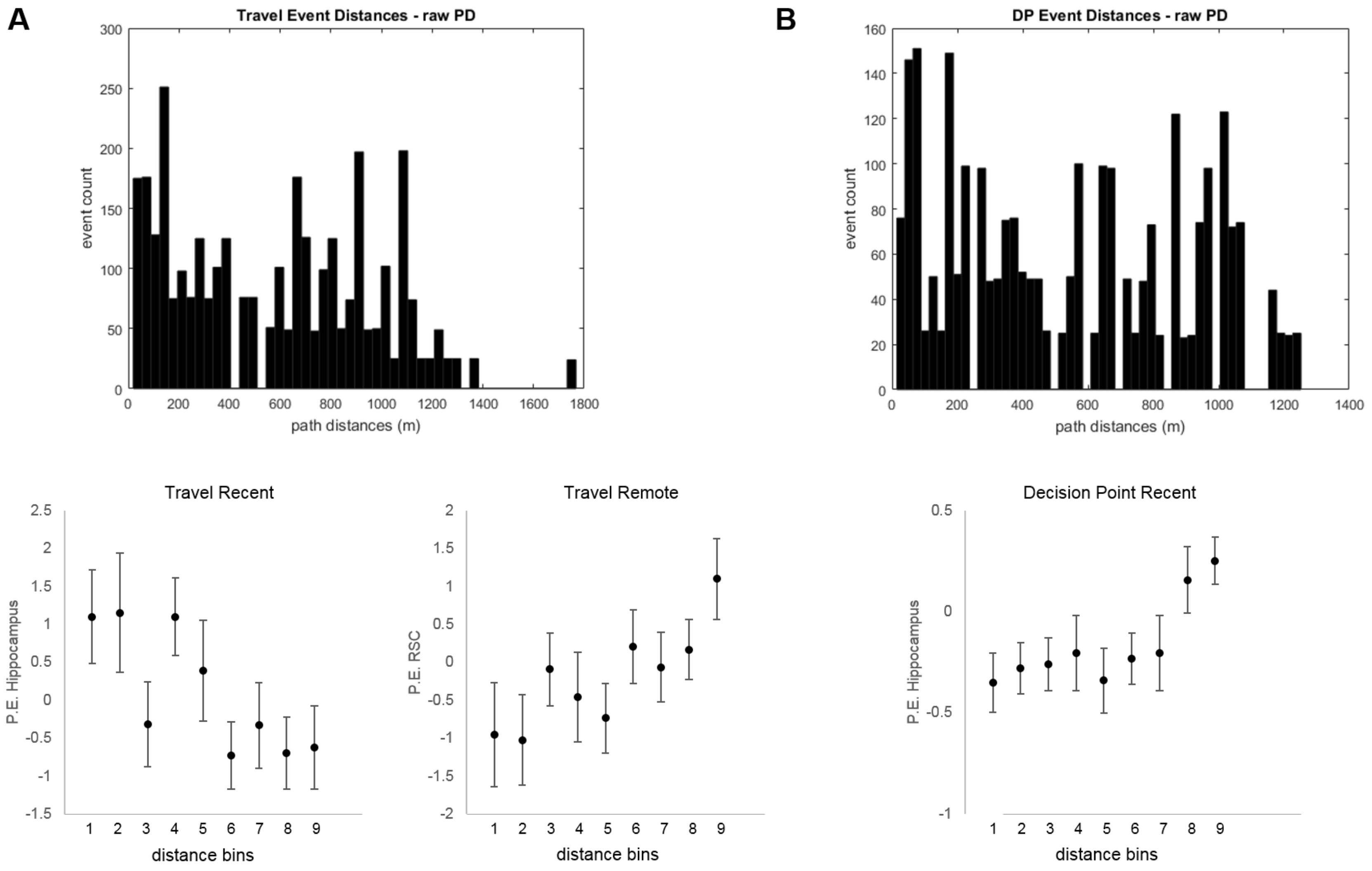

Figure S4: Detailed plot of linear effects by distance bin at Travel (A) and Decision Points (B). A: Top plot shows the range of path distances to the goal during Travel periods (across all subjects). Bottom: The results of Figure 2&3 replotted showing the linear effects in more detail. B: Same for Decision points, from Figure S5. Note these plots are for illustration purposes only.

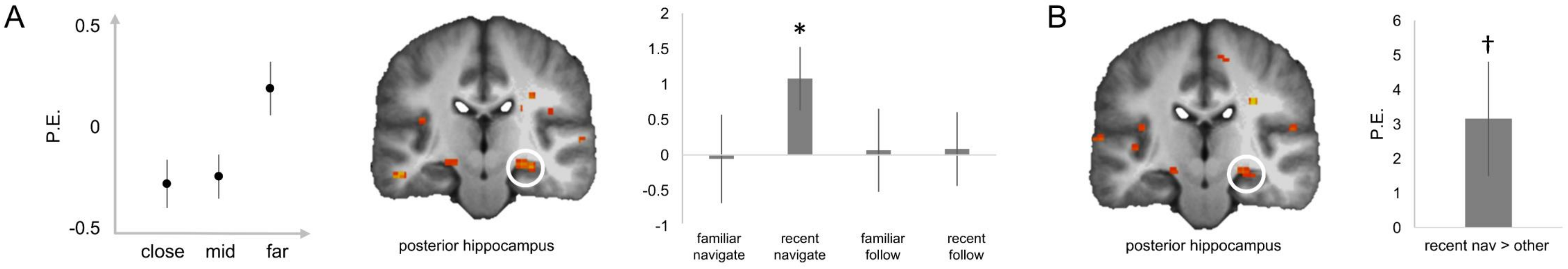

Figure S5: Path distance coding at Decision Points. A) At Decision Points, there was a significant positive correlation with path distance, such that there was higher BOLD activity in the right hippocampus when participants were further away from the goal location in recent environments. From left to right: parameter estimates (PE) extracted from a categorical model (binned by distance), the BOLD activity for the relevant condition (displayed at p<0.005, min. 5 contiguous voxels), and the PE from the peak voxel in the ROI for each condition, for illustrative purposes only (note the '*' refers to SVC of the voxels in the SPM). The effect plotted is corrected for IES (inverse efficiency), and is significant with and without this correction, thus underscoring that it is not a RT (or difficulty) effect. It also survives small-volume correction, including when ED (Euclidian distance) is added to the model. B) Brain activity when the GLM included weighted regressors for the effects seen in A. The contrast was -1 3 -1 -1, testing for an overall correlation with path distance during Decision Points, for the recent navigation condition. *p<0.05 SVC, †p=0.005 un corrected

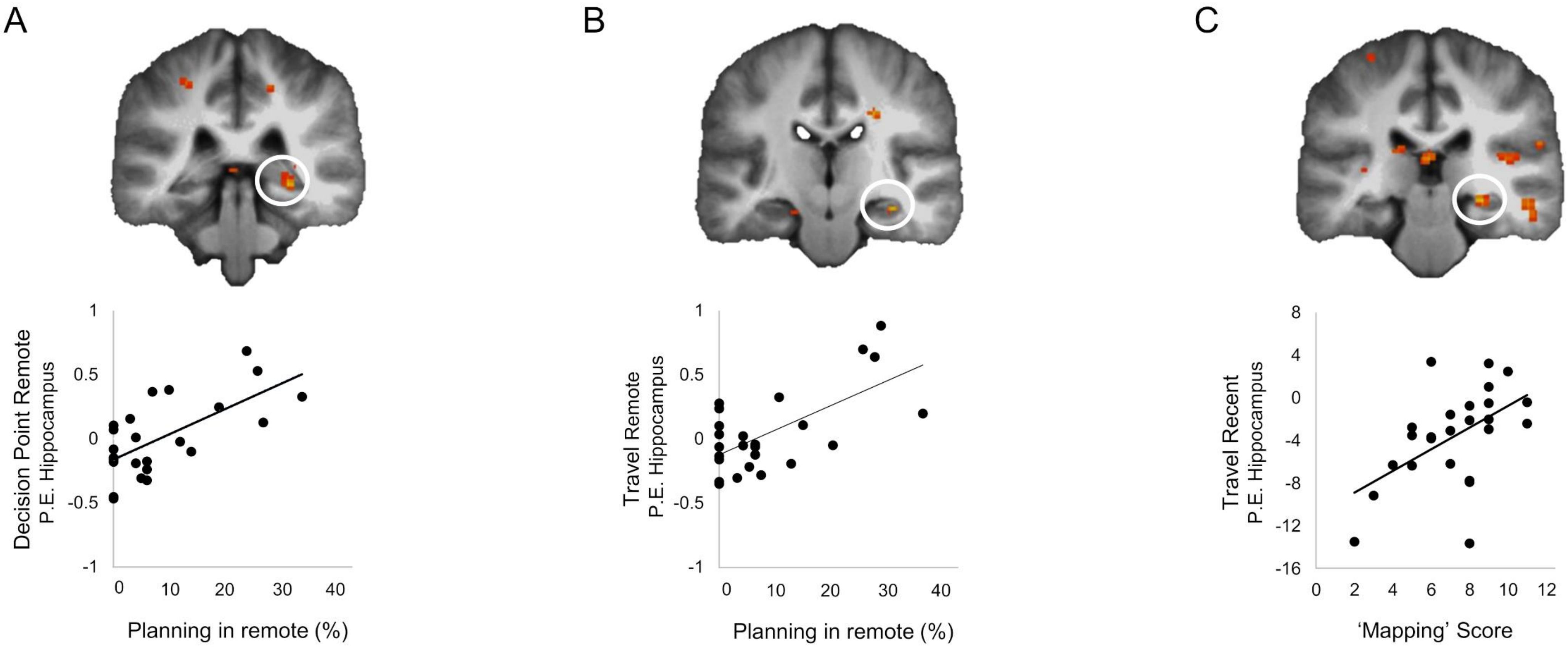

Figure S6: Right hippocampus active for people who plan and those who think in 'map-space'. A) People who report planning more when at Decision points in familiar environments show more right posterior hippocampal activity at Decision Points. B) People who report planning more when at Decision points in familiar environments show more right posterior hippocampal activity during Travel. Both A&B ($r>0.6, p<0.001$). C) Top: People who report using more map-like strategies during navigation have more posterior hippocampal activity in relation to path distance during Travel in recent environments. Bottom: Plotted is the same effect, but extracting parameter estimates per person in the peak hippocampal voxel from Fig3A, and correlating it with mapping scores ($r=0.53, p=0.007$). All other conditions did not significantly correlate with mapping scores ($r<.24$, $p>.1$).

Figure S7: Masks used of small-volume correction (SVC)
red: mid-posterior hippocampus; blue: retrosplenial cortex; yellow: remote memory

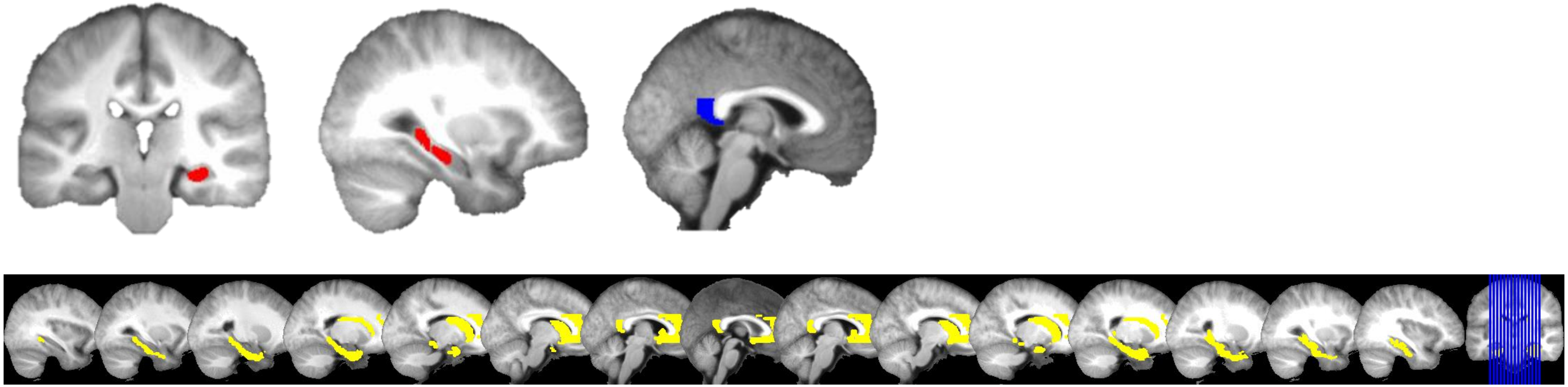

Supplement: Supplementary Data [file bhz044_supplement_materials.zip › bhz044_PATAI_CC_SuppMat.pdf]
